# Prognostic significance of neutrophil lymphocyte ratio and platelet lymphocyte ratio in advanced gastric cancer patients treated with FOLFOX chemotherapy

**DOI:** 10.1186/1471-2407-13-350

**Published:** 2013-07-22

**Authors:** Suee Lee, Sung Yong Oh, Sung Hyun Kim, Ji Hyun Lee, Min Chan Kim, Ki Han Kim, Hyo-Jin Kim

**Affiliations:** 1 Department of Internal Medicine, Dong-A University College of Medicine, 3-1 Dongdaeshin-dong, Busan, Seo-gu 602-715, Korea; 2 Department of Surgery, Dong-A University College of Medicine, Busan, Korea

**Keywords:** Neutrophil, Lymphocyte, Platelet, Gastric neoplasm

## Abstract

**Background:**

Several inflammatory response materials could be used for prediction of prognosis of cancer patients. The neutrophil lymphocyte ratio (NLR), and the platelet lymphocyte ratio (PLR) have been introduced for prognostic scoring system in various cancers. The objective of this study was to determine whether the NLR or the PLR would predict the clinical outcomes in advanced gastric cancer patients treated with oxaliplatin/ 5-fluorouracil (FOLFOX).

**Methods:**

The study population consisted of 174 advanced gastric cancer patients. Patients were treated with 85 mg/m^2^ of oxaliplatin as a 2-h infusion at day 1 plus 20 mg/m^2^ of leucovorin over 10 min, followed by 5-FU bolus 400 mg/m^2^ and 22-h continuous infusion of 600 mg/m^2^ at days 1-2. Treatment was repeated in 2-week intervals. The NLR and PLR were calculated from complete blood counts in laboratory test before and after first cycle of chemotherapy.

**Results:**

NLR was a useful prognostic biomarker for predicting inferior overall survival (OS) (*p* = 0.005), but was not associated with progression free survival (PFS) (*p* = 0.461). The normalization of NLR after one cycle of chemotherapy was found to be in association with significant improvement in PFS (5.3 months vs. 2.4 months, *p* < 0.001), and OS (11.9 months vs. 4.6 months, *p* < 0.001). The normalization of PLR was also associated with longer PFS (5.6 months vs. 3.4 months, *p* = 0.006), and OS (16.9 months vs. 10.9 months, *p* = 0.002). In multivariate analysis, changes in NLR were associated with PFS (Hazard ratio (HR): 2.297, 95% confidence interval (CI): 1.429-3.693, *p* = 0.001). The NLR, (HR: 0.245, 95% CI: 0.092-0.633, *p* = 0.004), PLR (HR: 0.347, 95% CI: 0.142-0.847, *p* = 0.020), changes in NLR (HR: 2.468, 95% CI: 1.567-3.886, *p* < 0.001), and changes in PLR (HR: 1.473, 95% CI: 1.038-2.090, *p* = 0.030) were independent prognostic markers for OS.

**Conclusion:**

This study demonstrates that NLR, PLR, and changes in NLR or PLR are independent prognostic factor for OS in patients with advanced gastric cancer treated with chemotherapy. These specific factors may also help in identifying the patients, who are more sensitive to FOLFOX regimen.

## Background

Gastric cancer remains a significant health problem despite its declining incidence in the West. It is the fourth most common cancer worldwide, accounting for 8.6% of all new cancer diagnoses in 2002 [1]. Although the incidence of stomach cancer among Korean has decreased over the past two decades, gastric cancer is the most common carcinoma in men, and the third most common type of cancer in women, and it remains the leading cause of death due to cancer in Korea [2].

Most of the newly diagnosed gastric patients present with either regional or distant metastatic disease where the 5-year overall survival is dismal and is generally accepted as being less than 10% [3]. Up to date, median survival beyond 12 months has not yet been achieved in any randomized study with combination chemotherapy [4]. The chemotherapeutic agent, 5-fluorouracil (5-FU) remains the main agent for the treatment of gastric cancer, and combination chemotherapy with 5-FU has demonstrated improved clinical outcomes. The 5-FU with cisplatin has shown an effective clinical outcome, however, the extent of toxicities were considerable [4]. Oxaliplatin, another platinum-based agent, has a more favorable tolerability profile than cisplatin. The oxaliplatin/5-FU combination (FOLFOX) has proven to be an effective first- or second-line treatment for advanced gastric cancer [5,6]. Increasing emphasis on the need for improved techniques for the prediction of treatment response and survival may facilitate the tailoring of chemotherapy and risk-related therapy, resulting in significantly better survival.

Although many biomarkers have been defined and studied in depth, excessive costs and technical factors often preclude their clinical use. Laboratory markers of systemic inflammation have been investigated as both prognostic and predictive biomarkers in several cancer populations. Assessment of the inflammatory response to the tumor may be easier and more-cost effective in clinical practice. Examples of these include CRP [7], Glasgow Prognostic Score (GPS) [8,9], neutrophil/lymphocyte ratio (NLR) [10,11] and platelet/lymphocyte ratio (PLR) [12,13] in predicting outcomes for patients after surgical resection but also in the patients with inoperable cancers.

There have been reports that a high density of neutrophils may actually promote tumor growth and metastasis [14] or suppress lymphocyte activity, thereby counteracting the antitumor immune response [15]. These observations suggest that an imbalance of NLR in the peripheral blood of cancer patients may be associated with tumor development. However, only limited information on the clinical significance and prognostic significance of NLR in patients with gastric cancer has been reported [9,16,17].

Thrombocytosis is caused by the stimulation of megakaryocytes by proinflammatory cytokines [18], and its association with prognosis shown in other related studies may be explained based on an elevated platelet count being an indicator of the severity of inflammation. The platelet count is another convenient parameter within the blood cell count that can help to predict patients’ survival. An increased PLR has been reported as an independent risk factor for reduced survival in pancreatic cancer or colorectal cancer [12,13]. The presence of both neutrophilia and thrombocytosis is likely to represent a nonspecific response to cancer-related inflammation and its associated release of cytokines. It is contemplated that neutrophilia compared with thrombocytosis is the most sensitive response, which best indicates the inflammatory activity of the tumor and causes a reduced survival through a mulifactorial process.

Evidence for the use of these inflammatory markers as direct predictors of outcome in patients with advanced malignancy receiving first-line chemotherapy is lacking. An elevated NLR in colorectal cancer patients with liver metastases receiving only neoadjuvant chemotherapy before surgical resection of liver metastases predicted worse rate of survival [11]. In addition, the patients in whom NLR normalized after one cycle of chemotherapy had significantly improved progression free survival similar to cases of advanced colorectal patients [10]. These data suggest that NLR may be a readily available and useful biomarker for monitoring early response and prognosis with chemotherapy. However, there was no report of significance of PLR to predict tumor response.

Therefore, we conducted the present study to evaluate the association of pretreatment levels of NLR or PLR with the clinical outcome of advanced gastric cancer patients, who were treated with FOLFOX chemotherapy. Moreover, it is imperative to clarify the impact of normalization of NLR or PLR for monitoring early response during chemotherapy.

## Methods

### Study population

All the patients in this study had histologically confirmed adenocarcinoma of the stomach. These patients were treated with FOLFOX chemotherapy. All the patients were aged between 18 and 79 years and had a performance status of less than or equal to two according to the Eastern Cooperative Oncology Group scale, and adequate bone marrow and renal function. The inclusion criteria included completion of previous adjuvant chemotherapy at least 6 months before inclusion. Exclusion criteria included the presence of central nervous system metastases, serious or uncontrolled concurrent medical illness, and a history of other malignancies. Written informed consent was obtained from each patient before study entry. The use of all patient material was approved by the institutional review board of Dong-A University Hospital.

### Treatment protocols and dose modification

On day 1, oxaliplatin (85 mg/m^2^) was administered by intravenous (i.v.) infusion in 500 ml of normal saline or dextrose over a period of 2 h. On day 1 and 2, leucovorin (20 mg/m^2^) was administered as an i.v. bolus, immediately followed by 5-FU (400 mg/m^2^) given as a 10-min i.v. bolus, followed by 5-FU (600 mg/m^2^) as a continuous 22-h infusion, with a light shield. Dose modifications of oxaliplatin or 5-FU were made for hematologic, gastrointestinal, or neurologic toxic effects based on the most severe grade of toxicity that had occurred during the previous cycle. Treatment could be delayed for up to 2 weeks if symptomatic toxicity persisted, or if the absolute number of neutrophils was <1,500/μl or platelets count was <100,000/μl. The dosage of 5-FU was reduced by 25% for subsequent courses after the occurrence of National Cancer Institute Common Toxicity Criteria (NCI-CTC) grade 3 diarrhea, stomatitis, or dermatitis. The dose of oxaliplatin was reduced by 25% in subsequent cycles if there were persistent paresthesias between cycles or paresthesias with functional impairment lasting >7 days. Treatment was continued until there were signs of disease progression, development of unacceptable toxic effects, or the patient refused further treatment.

### Follow-up evaluation and assessment of response

Before each treatment courses, a physical examination, routine hematology, biochemistry, and chest X-ray were carried out. Computed tomography scans to define the extent of the disease, and the responses were carried out after four cycles of chemotherapy, or sooner if there was evidence of any clinical deterioration. Patients were assessed before initiating each 2-week cycle using the NCI-CTC, except in the case of neurotoxicity. For the neurotoxicity, an oxaliplatin-specific scale was used: grade 1, paresthesias or dysesthesias of short duration, but resolving before the next dosing; grade 2, paresthesias persisting between doses (2 weeks); and grade 3, paresthesias interfering with function.

Responses were evaluated using RECIST criteria. Complete response (CR) was defined as the disappearance of all evidence of disease and the normalization of tumor markers for at least 2 weeks. Partial response (PR) was defined as ≥ 30% reduction in uni-dimensional tumor measurements, without the appearance of any new lesions or the progression of any existing lesion. Progressive disease (PD) was defined as any of the following: 20% increase in the sum of the products of all measurable lesions, appearance of any new lesion, or reappearance of any lesion that had previously disappeared. Stable disease (SD) was defined as a tumor response not fulfilling the criteria for CR, PR, or PD.

### Blood sample analysis

Venous blood samples were taken from patients admitted to the oncology outpatient clinic for palliative chemotherapy, and were collected in ethylene diamine tetraacetic acid (EDTA)-containing tubes. The exclusion criteria were history of blood transfusion within the last two months, active bleeding, bleeding diathesis, hyper- or hypothyroidism, infections, disseminated intravascular coagulation, heparin treatment or connective tissue disease.

WBC differential counts were analyzed by XE-2100 hematology analyzer (Sysmex, Kobe, Japan), and CEA were evaluated by Architect i2000 (Abbott Laboratories, USA). The NLR was calculated from the differential count by dividing the neutrophil measurement by the lymphocyte measurement. An NLR 3 or greater was considered as elevated. PLR was evaluated as platelet count divided by lymphocyte count. The calculated values were divided into two categories as <160 or ≥160. Both NLR and PLR were recorded at baseline and where available after 1 cycle of systemic therapy.

### Statistical analysis

The associations between NLR or PLR and the clinicopathologic parameters (sex, age, CEA, tumor size, differentiation, depth of bowel wall invasion, number of positive lymph nodes, vascular invasion) were assessed via χ^2^ or Fisher’s exact tests.

The progression free survival (PFS) and overall survival (OS) were calculated from the date of initiation of therapy to the date of disease progression and death, respectively. Patients who were alive at the last follow-up were censored at that time. Patients, who were taken off from the study or who died before progressions were censored at the time when they were taken off from the study. The association of each marker with survival was analyzed using Kaplan–Meier plots, the log-rank test, and its associated 95% confidence interval (CI) was calculated. Multivariate analyses were carried out using the Cox proportional hazards model. Variables with *p* < 0.10 on univariate analysis were entered into multivariate analyses. All the tests were two-sided, and *p* < 0.05 was considered statistically significant. Analyses were done using SPSS version 19.0 (SPSS Inc, Chicago, IL).

## Results

### Patient characteristics

From March 2007 to August 2010, a total of 174 patients enrolled in the present study. The median follow-up time was 14.9 (range 1.0-47.9 months) months. Demographic details about the patients included in the present study are shown in Table 1. Overall, there were 110 (65.5%) male and 64 (34.5%) female patients, and the median age was 55 ±12.4 years (range 24–74). One hundred and sixteen (66.7%) patients underwent operation. Among them, seventy-four (42.5%) patients received 5-FU-based adjuvant chemotherapy. All the patients had ECOG performance status of zero or 1. None of the patients showed clinical signs of sepsis or other inflammatory illnesses at the time of commencement of systemic therapy.

**Table 1:** Patients’ characteristics

	**Number**	
Age		
< 60 years	109	62.6%
≥ 60 years	65	37.4%
Gender		
Male	114	65.5%
Female	60	34.5%
Previous operation		
+	116	66.7%
-	58	33.3%
Initial TNM stage		
I	7	4.0%
II	22	12.6%
III	41	23.6%
IV	104	59.8%
Lauren’s classification		
Diffuse	40	23.0%
Intestinal	25	14.4%
Mixed	94	54.0%
Unknown	15	8.6%
Adjuvant chemotherapy		
+	74	42.5%
-	100	57.5%
CEA		
< 5 ng/ml	118	67.8%
≥ 5 ng/ml	56	32.2%
Number of metastasis		
1	95	54.6%
> 1	79	45.4%
ECOG Performance status		
0-1	100	100%

*CEA *Carcinoembryonic antigen, *ECOG *Eastern cooperative oncology group.

### Prognostic variables according to NLR and PLR

The median neutrophil count was 3.93 × 10^6^/ml (range 3.01-20.34), and lymphocyte count was 1.62 × 10^6^/ml (range 0.51-20.92). Correlations between the NLR and clinicopatholotic parameters are shown in Table 2. The NLR was grouped with respect to 2 different cutoff points (≥ 3 or < 3). One hundred and twelve patients (64.4%) were detected with NLR of less than 3, while there were 62 patients (35.6%) whose NLR was greater than or equal to 3. No significant correlations were noted between NLR and gender, age, or CEA level. The association between NLR and previous operation (*p* = 0.002), and number of metastatic sites (*p* = 0.027) were statistically significant.

**Table 2:** Association of neutrophil lymphocyte ratio with patients’ characteristics

	**NLR < 3**		**NLR ≥ 3**		***p***
	**No.**	***%***	**No.**	***%***	
Age					0.624
< 60 years	72	66.1	37	33.9	
≥ 60 years	40	61.5	25	38.5	
Gender					0.136
Male	78	68.4	36	31.6	
Female	34	56.7	26	43.3	
Previous operation					0.002
+	84	72.4	32	27.6	
-	28	48.3	30	51.7	
Adjuvant chemotherapy					0.004
+	57	77.0	17	23.0	
-	55	55.0	45	45.0	
Lauren’s classification					0.014
Diffuse	28	70.0	12	30.0	
Intestinal	21	84.0	4	16.0	
Mixed	51	54.3	43	45.7	
Unknown	12	80.0	3	20.0	
CEA					1.000
< 5 ng/ml	76	64.4	42	35.6	
≥ 5 ng/ml	36	64.3	20	35.7	
Number of metastasis					0.027
1	54	56.8	41	43.2	
> 1	58	73.4	21	26.6	

*NLR *Neutrophil lymphocyte ratio, *CEA *Carcinoembryonic antigen.

The median value of platelet was observed to be 263 × 10^6^/ml (range 189-872). Table 3 summarizes the patient characteristics at baseline according to PLR. The PLR was grouped on the basis of 2 different cutoff points (≥ 160 or < 160). About 88 patients (50.6%) were detected with PLR less than 160, while there were 86 patients (49.4%), whose PLR was greater than or equal to 160. Moreover, PLR was found to be in significant correlation with gender (*p* = 0.011), previous operation (*p* = 0.004), and adjuvant chemotherapy (*p* < 0.001). The PLR less than 160 was found to be in association with lower NLR (< 3) value (*p* < 0.001).

**Table 3:** Association of platelet lymphocyte ratio with patients’ characteristics

	**PLR < 160**		**PLR ≥ 160**		***p***
	**No.**	***%***	**No.**	***%***	
Age					0.756
< 60 years	54	49.5	55	50.5	
≥ 60 years	34	52.3	31	47.7	
Gender					0.011
Male	66	57.9	48	42.1	
Female	22	36.7	38	63.3	
Previous operation					0.004
+	68	58.6	48	41.4	
-	20	34.5	38	65.5	
Adjuvant chemotherapy					< 0.001
+	49	66.2	25	33.8	
-	39	39.0	61	70.9	
Lauren’s classification					0.078
Diffuse	20	50.0	20	50.0	
Intestinal	14	56.0	11	44.0	
Mixed	42	44.7	52	55.3	
Unknown	12	80.0	3	20.0	
CEA					0.195
< 5 ng/ml	64	54.2	54	45.8	
≥ 5 ng/ml	24	42.9	32	57.1	
Number of metastasis					0.070
1	42	44.2	53	55.8	
> 1	46	58.2	33	41.8	

*CEA *Carcinoembryonic antigen, *PLR *Platelet lymphocyte ratio.

### Association of NLR or PLR with chemotherapy response

The median number of cycles of FOLFOX chemotherapy was 5 (range 2-23). The overall response rate was 36.8%, while stable disease was 39.1%. Table 4 shows the association of patients’ clinicopathologic features with chemotherapy response. The features, gender (*p* = 0.049), and Lauren’s classification (*p* = 0.042) were found to be related to the response to chemotherapy. Male or intestinal type was found to be associated with better response to FOLFOX chemotherapy. Other parameters, such as age, previous operation, and CEA level were not found to be in significant correlation with clinical response. We analyzed the association of pretreatment NLR, PLR, and changes in NLR or PLR after 1 cycle of chemotherapy with tumor response to FOLFOX chemotherapy. None of the markers was significantly correlated with response.

**Table 4:** Prognostic factors in univariate analysis

	**Response rate (%)**	***p***	**TTP (months)**	***p***	**OS (months)**	***p***
Age		1.000		0.002		0.015
< 60 years	36.7		5.1		16.0	
≥ 60 years	36.9		3.9		10.2	
Gender		0.049		0.148		0.117
Male	42.1		4.8		13.9	
Female	26.7		4.1		12.4	
Previous operation		0.068		0.173		< 0.001
+	31.9		4.6		15.8	
-	46.6		4.6		10.5	
Lauren’s classification		0.042		0.194		0.157
Diffuse	20.0		4.1		13.1	
Intestinal	48.0		6.4		19.9	
Mixed	42.6		4.7		11.5	
Unknown	26.7		3.9		13.3	
Adjuvant chemotherapy		0.205		0.655		0.181
+	31.1		4.6		12.9	
-	41.0		4.8		13.2	
CEA		0.737		0.976		0.154
< 5 ng/ml	35.6		4.4		15.1	
≥ 5 ng/ml	39.3		4.6		11.5	
Number of metastasis		0.430		0.276		0.335
1	33.7		4.2		13.2	
> 1	40.5		4.6		13.1	
NLR		0.327		0.461		0.005
< 3	33.9		4.6		15.8	
≥ 3	41.9		4.6		10.9	
PLR		0.530		0.285		0.098
< 160	34.1		4.9		13.3	
≥ 160	39.5		4.0		12.2	
cNLR		0.349		< 0.001		< 0.001
< 3 → <3	35.1		4.9		17.3	
<3 → ≥ 3	26.7		3.1		8.6	
≥ 3 → < 3	37.5		5.3		11.9	
≥ 3 → ≥ 3	57.1		2.4		4.6	
cPLR		0.757		0.006		0.002
< 160 → <160	36.4		4.9		13.3	
<160 → ≥160	27.3		5.3		10.6	
≥ 160→ <160	38.2		5.6		16.9	
≥ 160 → ≥ 160	40.4		3.4		10.9	

*CEA* Carcinoembryonic antigen, *NLR* Neutrophil lymphocyte ratio, *PLR* Platelet lymphocyte ratio, *cNLR* Change of neutrophil lymphocyte ratio after 1 cycle of chemotherapy, *cPLR* Change of platelet lymphocyte ratio after 1 cycle of chemotherapy.

### Association of NLR or PLR with survival

**Figure 1:**
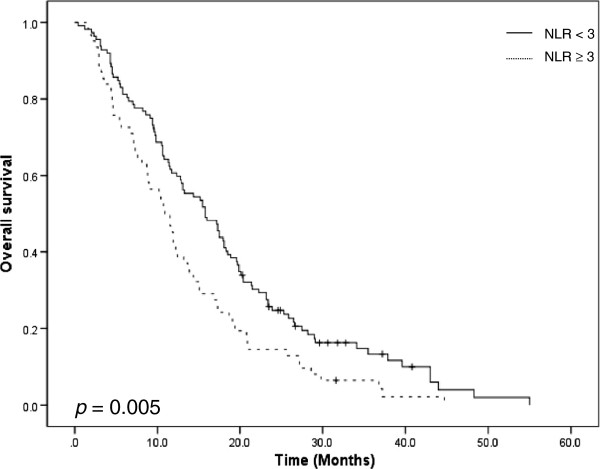
**Overall survival curve according to NLR. **NLR: neutrophil to lymphocyte ratio.

The median PFS was 4.2 months (95% CI: 3.5-4.8 months), and the median OS was 13.1 months (95% CI: 10.6-15.5 months). The results of univariate analysis for the predictors of survival are listed in Table 4. Univariate analysis revealed that old age was a predictor of worse PFS (*p* = 0.002). Other parameters were not found to be in correlation with PFS. Age (*p* = 0.015), previous operation (*p* < 0.001), and NLR (*p* = 0.005) were found to be significantly associated with OS. Patients with NLR ≥ 3 showed shorter OS than patients with NLR of less than 3 (10.9 vs. 15.8 months, *p* = 0.005; Figure 1).

**Figure 2:**
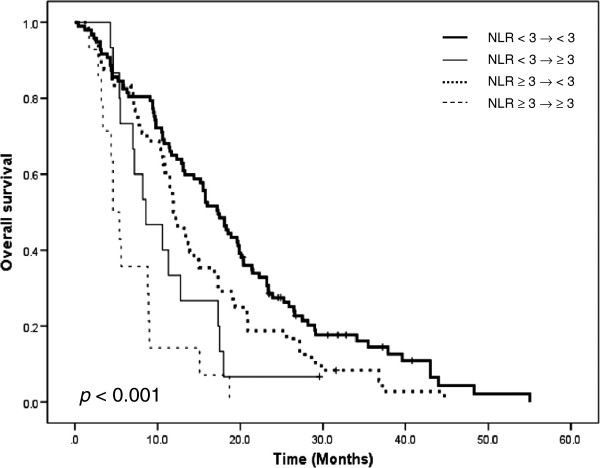
**Overall survival curve according to change of NLR after 1 cycle of chemotherapy.** NLR: neutrophil to lymphocyte ratio.

Patients were categorized into 4 groups according to the changes in NLR after first cycle of chemotherapy. (1) NLR < 3 at baseline and after 1 cycle of chemotherapy (n = 97, cohort 1), (2) NLR < 3 at baseline and ≥ 3 after 1 cycle of chemotherapy (n = 15, cohort 2), (3) NLR ≥ 3 at baseline with normalization of NLR < 3 after 1 cycle of chemotherapy (n = 48, cohort 3), and (4) NLR ≥ 3 at baseline and after 1 cycle of chemotherapy (n = 14, cohort 4). Patients with lower NLR before 2^nd^ cycle of chemotherapy (cohort 1, 3) had an improved PFS when compared with patients with higher NLR (cohort 2, 4; *p* < 0.001). Normalization of NLR led to an improvement in median OS from 4.6 months (cohort 4) to 11.9 months (cohort 3) in patients with persistently elevated NLR (*p* < 0.001; Figure 2).

**Figure 3:**
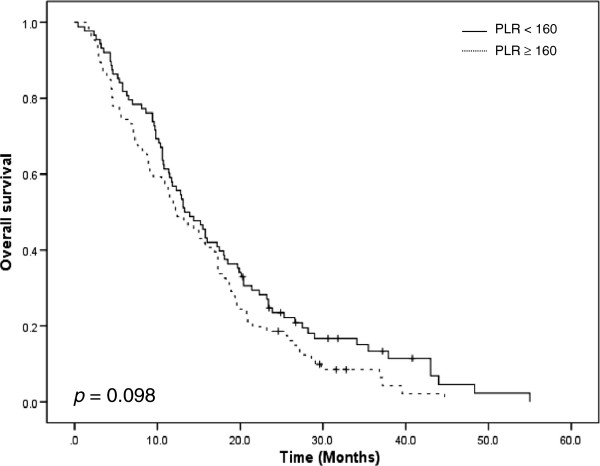
**Overall survival curve according to PLR. **LR: platelet to lymphocyte ratio.

**Figure 4:**
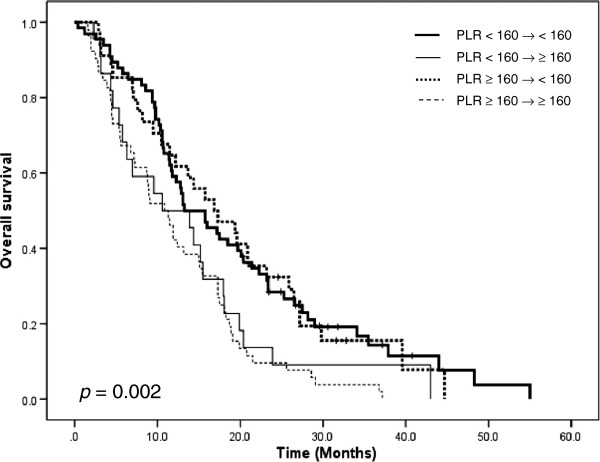
**Overall survival curve according to change of PLR after 1 cycle of chemotherapy.** PLR: platelet to lymphocyte ratio.

The PLR did not demonstrate a significant relationship with OS (p=0.098; Figure 3), although there was an inclination towards a shorter survival when the PLR was ≥ 160 (13.3 months) compared with less than 160 (12.2 months). Kaplan-Meier cumulative survival curve for patients stratified with PLR groups are shown in Figure 4. Patients were categorized into 4 groups according to the changes in PLR after first cycle of chemotherapy. (1) PLR < 160 at baseline and after 1 cycle of chemotherapy (n = 66, cohort 1), (2) PLR < 160 at baseline and ≥ 160 after 1 cycle of chemotherapy (n = 22, cohort 2), (3) PLR ≥ 160 at baseline with normalization of PLR < 160 after 1 cycle of chemotherapy (n = 34, cohort 3), and (4) PLR ≥ 160 at baseline and after 1 cycle of chemotherapy (n = 52, cohort 4). Patients with PLR equal or higher than 160 before and after 1 cycle of chemotherapy (cohort 4) were the worst PFS when compared with other cohort (p = 0.006). Normalization of PLR improved median OS from 10.9 months (cohort 4) to 16.9 months (cohort 3) in patients with persistently elevated PLR (*p* = 0.002; Figure 4).

In order to assess the independent prognostic factor, we utilized multivariate Cox proportional hazard analysis as a control for other prognostic values. In multivariate analysis, age (Hazard ratio (HR): 1.655, 95% Confidence Interval (CI): 1.180-2.322, *p* = 0.004), and changes in NLR (HR; 2.297, 95% CI: 1.429-3.693, *p* = 0.001) were found to be associated with PFS. Age (HR: 1.412, 95% CI: 1.016-1.961, *p* = 0.040), previous operation (HR: 1.641, 95% CI: 1.145-2.351, *p* = 0.007), NLR (HR: 2.245, 95% CI: 2.092-3.633, *p* = 0.004), PLR (HR: 1.743, 95% CI: 1.142-2.847, *p* = 0.020), changes in NLR (HR: 2.468, 95% CI: 1.567-3.886, *p* < 0.001), and changes in PLR (HR:1.473, 95% CI: 1.038-2.090, *p* = 0.030) were independent prognostic markers for OS (Table 5).

**Table 5:** Multivariate analysis

	**Overall survival**		
	**HR**	**95% CI**	***p***
Age	1.412	1.016 - 1.961	0.040
Previous operation	1.641	1.145 - 2.351	0.007
NLR	2.245	2.092 - 3.633	0.004
PLR	1.743	1.142 - 2.847	0.020
cNLR	2.468	1.567 - 3.886	< 0.001
cPLR	1.473	1.038 - 2.090	0.030

*NLR *Neutrophil lymphocyte ratio, *PLR *Platelet lymphocyte ratio, *cNLR *Change of neutrophil lymphocyte ratio after 1 cycle of chemotherapy, *cPLR *Change of platelet lymphocyte ratio after 1 cycle of chemotherapy.

## Discussion

The FOLFOX regimen is used as an effective palliative treatment for gastric cancer [5,6]. Previously, we have reported on the effectiveness of oxaliplatin with biweekly low-dose leucovorin and bolus/continuous infusion of 5-FU (modified FOLFOX 4) as a first line therapy in advanced gastric cancer patients, and found a response rate of 50.0%, a median TTP of 7.7 months, and a median OS time of 11.2 months [5].

Despite a short overall survival, great heterogeneity exists in the length of survival among patients. Several serum and tissue molecular markers have previously been analyzed as candidate predictors of chemosensitivity. We have also reported that immunohistochemical staining for ERCC1 may be useful in prediction of the clinical outcome in advanced gastric cancer patients treated with modified FOLFOX4 [19]. It has also been shown that the GSTM1 positive genotype evidenced a significantly better time to progression in cases of advanced gastric cancer being treated with FOLFOX [20]. However, none of these factors is currently used clinically because of the complex methodology and low accuracy of prediction.

The immune response triggered by cancer is very complex in nature. The presence of T cells in tumor indicates significant immune response to the lesion [21]. Lymphocytopenia induced by the systemic inflammatory response reveals depression of innate cellular immunity indicated by a marked decrease in T4 helper lymphocytes and an increase in T8 suppressor lymphocytes [22]. Alternatively, neutrophilia may aid in the development and progression of the cancer by providing an adequate environment for its growth. Circulating neutrophils have been shown to contain and secrete the majority of circulating vascular endothelial growth factor that is thought to play a pivotal role in tumor development [23]. Hence, a high density of circulating neutrophils may exert unfavorable effects on the tumor-bearing host, thus leading to a negative correlation between neutrophil density and patient survival. NLR can be considered as the balance between pro-tumor inflammatory status and anti-tumor immune status. Patients with elevated NLR have a relative lymphocytopenia and neutrophilc leukocytosis, which denotes that the balance is tipped in favor of pro-tumor inflammatory response and is associated with poor oncologic outcome [10,11,16,17].

Previously, there has been a report regarding the significant correlation between the NLR and survival in the patients with Stage IV gastric cancer [16]. The authors included patients who had either histologically or cytologically confirmed Stage IV gastric cancer and were treated with S-1 monotherapy without operation. The authors inferred that in patients with NLR value of less than 2.5, the median survival time was significantly higher than the ones with NLR of > 2.5 like in advanced gastric cancer (363 days vs. 239 days). Similarly, in the present study, the patients with an NLR value lower than 3 had significantly higher median duration of survival than those with an NLR value of 3 or above (15.9 months. vs. 10.9 months, p = 0.005). The cutoff value used in the present study was different from in the ones used in previous reports [10,11,16,17]. We analyzed OS curves according to each NLR value; based on univariate analysis of these survival curves, 3.0 was the best cutoff value to distinguish patients with a poor prognosis from those with a good prognosis.

We also found that high NLR reverted to normal after 1 cycle of chemotherapy in 48 patients (27.6%). The survival of these 48 patients was 11.9 months, and was better than of the patients who’s NLR remained high (4.6 months). The survival of the 48 patients with normalized NLR was also better than the patients whose NLR was increased from < 3 to ≥ 3 (11.9 months. vs. 8.6 months). This finding confirms the important role of NLR in predicting survival and provides an early marker both to predict outcome in advanced gastric cancer patients with an initial abnormal NLR and in assisting clinical decision-making.

Platelet count is an additional index of systemic inflammation elicited by the tumor. Platelet aggregation and degranulation along with the consequent release of platelet-derived proangiogenic mediators within the microvasculature of the tumor also could be an important determinant of tumor growth [24]. No clear picture is available on the significance of tumor-platelet interactions. A number of proinflammatory mediators are known to stimulate megakaryocyte proliferation [18]. Smith et al. have defined preoperative PLR as an independent significant prognostic marker in resected pancreatic ductal adenocarcinoma [12]. We have previously reported that the median overall survival rate in patients with a PLR of 150 or less was 80.6%, 59.0% in the ones with a PLR of 151–300, and 53.9% in patients with a PLR greater than 300 in colorectal cancer patients underwent curative resection [13].

In the present study, for cases where PLR was less than 160 (n = 88), the median survival time was found to be 13.3 months. Nonetheless, the median survival interval was observed to be 12.2 months in patients whose PLR was higher than or equal to 160. However, this difference was not significant (*p* = 0.098). The patients, in whom PLR was normalized after 1 cycle of systemic therapy were found to have significantly longer survival than those whose PLR remained abnormal with a survival difference of 6 months (*p* = 0.002). This data would provide additional prognostic information for clinicians at an earlier time point before conventional evaluation of chemotherapy response using computed tomography scans and potentially identify a proportion of patients in whom further treatment may be futile.

To the best of our knowledge, this is the first study to describe the use of NLR and PLR in advanced gastric cancer patients receiving first-line palliative chemotherapy in terms of providing useful information regarding prognostication. This study also reports the investigation of the utility of NLR and PLR during the course of chemotherapy, in particular the normalization of NLR and PLR, to predict early responses to treatment. In this cohort, a subset of patients with normalization of NLR and PLR after first cycle of FOLFOX chemotherapy had substantially improved OS when compared to the patients without normalization of NLR and PLR. The NLR and PLR can be calculated from the data that are already routinely available. It does not require any additional expenditure. Moreover, hematologic markers are much cheaper and faster laboratory parameters, which can be measured than conventional tumor markers such as serum CEA, CA 19-9, and CA 72-4. The NLR and PLR can be used to routinely evaluate blood chemistry parameters for outpatients because of their lower cost and greater convenience in comparison with complex and expensive techniques such as computed tomography, magnetic resonance imaging, and positron emission tomography.

## Conclusions

In conclusion, we found that pretreatment routine hematological parameters including NLR and PLR were correlated with prognosis in patients with gastric cancer who had been treated with FOFLOX. Although this study was a retrospective analysis and a single-center study, it indicates the potential usefulness of a new predictor of the pathologic response to chemotherapy. The low cost and easy accessibility and reproducibility of a full blood count are other features promoting its use in clinical practice. To confirm these findings, larger, prospective, randomized studies are required in future.

## Competing interests

The authors declare that they have no competing interests.

## Authors’ contributions

SL collected the data, performed the statistical analysis and drafted the manuscript. SYO collected the data, performed the statistical analysis with interpretation and critically revised the manuscript. SHK and JHL performed the chemotherapy for patients and revised the manuscript. MCK and KHK performed the operations for patients and revised the manuscript. HJK conceived of the study, and approved the final manuscript. All authors read and approved the final manuscript.

## Pre-publication history

The pre-publication history for this paper can be accessed here:

http://www.biomedcentral.com/1471-2407/13/350/prepub
